# The Neuroendocrine Neoplasms of the Digestive Tract: Diagnosis, Treatment and Nutrition

**DOI:** 10.3390/nu12051437

**Published:** 2020-05-15

**Authors:** Jakub Pobłocki, Anna Jasińska, Anhelli Syrenicz, Elżbieta Andrysiak-Mamos, Małgorzata Szczuko

**Affiliations:** 1 Department of Endocrinology, Metabolic Diseases and Internal Diseases, Pomeranian Medical University in Szczecin, Unii Lubelskiej 1str, 70-252 Szczecin, Poland; jakub.poblocki@pum.edu.pl (J.P.); klinendo@pum.edu.pl (A.S.); Elamamos@o2.pl (E.A.-M.); 2 Department of Human Nutrition and Metabolomic, Pomeranian Medical University in Szczecin, Broniewskiego 24 str, 71-460 Szczecin, Poland; a.jas@wp.pl

**Keywords:** gastroenteropancreatic neuroendocrine neoplasms (GEP-NENs), neuroendocrine neoplasms (NEN), neuroendocrine tumors, biogenic amines, nutrition, therapy

## Abstract

Nuroendocrine neoplasms (NENs) are a group of rare neoplasms originating from dispersed neuroendocrine cells, mainly of the digestive and respiratory tract, showing characteristic histology and immunoprofile contributing to classification of NENs. Some NENs have the ability to produce biogenic amines and peptide hormones, which may be associated with clinical syndromes like, e.g., the carcinoid syndrome caused by unmetabolized overproduced serotonin, hypoglycemic syndrome in case of insulinoma, or Zollinger-Ellison syndrome accompanying gastrinoma. Diagnostics for these include ultrasound with endoscopic ultrasound (EUS), computed tomography (CT), magnetic resonance imaging (MRI), and positron-emission tomography/computed tomography (PET/CT). Different nuclear medicine procedures can also be used, like somatostatin analogues scintigraphy (SRS) and 68Ga-Dota-Peptide PET/CT, as well as biochemical methods to determine the level of general neuroendocrine markers, such as chromogranin A (CgA), 5-hydroxyindolacetic acid (5-HIAA), synaptopfysin and cell type-specific peptide hormones, and neurotransmitters like gastrin, insulin, serotonin, and histamine. NENs influence the whole organism by modulating metabolism. The treatment options for neuroendocrine neoplasms include surgery, somatostatin analogue therapy, radionuclide therapy, chemotherapy, molecular targeted therapies, alpha-interferon therapy, and inhibitors of serotonin production. In the case of hypersensitivity to biogenic amines, a diet that limits the main sources of amines should be used. The symptoms are usually connected with histamine, tyramine and putrescine. Exogenic sources of histamine are products that take a long time to mature and ferment. Patients with a genetic insufficiency of the diamine oxidase enzyme (DAO), and those that take medicine belonging to the group of monoamine oxidases (MAO), are particularly susceptible to the negative effects of amines. Diet plays an important role in the initiation, promotion, and progression of cancers. As a result of the illness, the consumption of some nutrients can be reduced, leading to nutritional deficiencies and resulting in malnutrition. Changes in metabolism may lead to cachexia in some patients suffering from NENs. The aim of this narrative review was to advance the knowledge in this area, and to determine possibilities related to dietary support. The authors also paid attention to role of biogenic amines in the treatment of patients with NENs. We can use this information to better understand nutritional issues faced by patients with gastroenteropancreatic neuroendocrine neoplasms (GEP-NENs), and to help inform the development of screening tools and clinical practice guidelines.

## 1. Introduction

Neuroendocrine neoplasms (NENs) are a heterogenic group of tumors originating from the endocrine glands (adrenal glands, pituitary gland, parathyroid glands), endocrine cells within gland tissues (pancreas, thyroid), or dispersed endocrine cells of the digestive and respiratory tracts [1]. The term neuroendocrine neoplasm is a general term which includes a group of well-differentiated neoplasms called neuroendocrine tumors (NETs), and a group of poorly differentiated forms called neuroendocrine cancers (NECs) [2].

The characteristic feature of some NENs is the ability to produce, store and secrete biogenic amines and peptide hormones, such as insulin, gastrin, vasoactive intestinal peptide, glucagon, or somatostatin [3,4,5,6,7,8,9].

Epidemiological NEN data show the incidence rate is about 5.6/100.000 per year [6]. NENs originating in the digestive system are called gastro-entero-pancreatic neuroendocrine neoplasms and represent 62–67% of NENs [7]. Small bowel NENs are the most common type of gastroenteropancreatic neuroendocrine neoplasms (GEP-NENs) originating from midgut and constituting about 38% of all GEP-NENs [10,11].

The occurrence of tumors within the small intestine, particularly the ileum, is estimated at about 2.8 to 8 cases per 1 million people each year. The incidence of neuroendocrine neoplasms of the duodenum is about 0.1 cases per 100,000 people, whereas, for the colon and the rectum, the values are respectively 7.8% and 13.7% of all NENs. The occurrence of stomach NEN does not exceed 2 cases per 100,000 people, whereas the value for the pancreas is about 4 to 12 cases per 1 million people per year [12].

NENs can originate everywhere in the body, not only in the gastrointestinal tract, pancreas, and lungs, but also in less frequent sites, such as the thymus, central nervous system, thyroid, skin, breast, and urogenital system [2]. The main location of NENs near the ileocecal valve often results in liver metastasis, which worsens the prognosis; the 10-year survivability decreases from 60% to 15–25%. The 5-year survivability of small intestine NETs is 73.8% in the case of local changes, but only 43.2% if distant metastasis occurs [12]. Moreover, malnutrition influences quality of life but also reduces tolerance to anti-cancer therapy and reduces survival in patients with cancer [13]. Currently, nutritional and vitamin status is a neglected area in patients with GEP-NENs [14]. Clinical practice guidelines and consensus guidelines for GEP-NENs with regards to best practice for diagnosis, treatment, and medical management are available, but the supportive care needs and optimal nutritional management of patients affected by these unique tumors remain under-researched [15]. The aim of this narrative review was to broaden and systematize knowledge in this area, determine the possibilities for dietary support and draw attention to the need for an anti-cancer diet rich in plant products and fiber. The aim was also to reduce along with the need to reduce at the same time for patients suffering from persistent diarrhea.

The authors paid attention to the role of alimentary biogenic amines in the genesis of general symptoms in the group of NENs patients, especially in those experiencing hypersensitivity to biogenic amines, and also focused on the different nutritional needs according to the severity of the disease and the patient’s nutritional status.

We hope that our manuscript will contribute to the development of screening tools and clinical practice guidelines.

### 1.1. NEN Diagnostics

NEN diagnostics should include: biochemical measurements, imaging diagnostics and histopathological examination, which are crucial to diagnose and classify NEN.

For many years, the measurement of serotonin metabolites such as 5-hydroxyindoleacetic acid (5-HIAA) in 24 h urine collection was sufficient for most purposes, and has been considered the best method in laboratory diagnostics for functioning NENs with carcinoid syndrome [16]. Unfortunately, this method has limitations, and the consumption of pineapples, bananas, eggplant, the common walnut, paracetamol, caffeine, and naproxen can lead to false positive results, while, on the other hand, in patients treated with acetylsalicylic acid, adrenocorticotropin, levodopa and phenothiazine derivatives, false negative results can occur. There is a possibility of including the serum, platelet and urine serotonin concentrations as well.

A significant NEN diagnostic method is measuring chromogranins, particularly chromogranins A and B, which are proteins created and secreted by neuroendocrine tissues, fulfilling the role of non-specific NEN markers [17]. Chromogranin A is associated with the size of the tumor and allows for a better evaluation (in comparison to 5-HIAA) of recurrence in patients with a diagnosed NEN. It has been demonstrated that CgA levels in the plasma correlate with the load of the tumor and predict the survivability of patients with small intestine NENs. However, there is no correlation between the CgA plasma and the weight of the tumor, or the survivability of colorectal NENs [18], whereas high mRNA-binding protein 3 (IMP3) expression levels were determined to be associated with a high disease stage in patients with GEP-NENs [19].

In imaging diagnostics, somatostatin receptor scintigraphy (SRS) can still be considered [20]. The main factors that support this method include the fact that the dominating subtypes of the receptor on the GEP-NEN cells, SSR2 (somatostatin receptor type 2) and SSR5 (somatostatin receptor type 5), are the main point of uptake of the activity of octreotide (somatostatin analogue), and the presence of somatostatin receptors on the surface of 80–90% of GEP-NEN cells. SRS provides information on the location of the tumor as well as the degree of its development, and it allows the response to treatment with somatostatin analogues to be determined [20,21,22].

Because of greater diagnostic accuracy and lower radiation dose in an increasing number of centers, PET/CT with 68Ga-labeled somatostatin analogues has replaced SRS [22]. Other methods, such as ultrasound, colonoscopy, gastroscopy, endosonography, computed tomography (CT), magnetic resonance imaging (MRI), and various kinds of positron emission tomography (PET/CT) like F-FDG_PET/CT or the before mentioned 68Ga-DOTA-Peptide PET/CT, are available and useful for the localization of NENs, as well as for staging [23,24,25].

According to World Health Organization (WHO) 2017, based on WHO 2010 and European Neuroendocrine Tumor Society (ENETS) classifications that are crucial for the diagnosis of NENs, the histopathological examination should be supplemented with immunohistochemistry, which is based on Ki67 expression and allows for the division of NENs into three main groups of well differentiated tumors: NET G1 (low grade) with Ki67 ≤ 2%, NET G2 with Ki67 3–20% (intermediate grade), and NET G3 with Ki67 > 20% (high grade). Additionally, analysis of the morphology of the NEN cells is essential, in separate wells, for poorly differentiated NENs. Poorly differentiated NENs with Ki67 > 20% are called neuroendocrine cancers (NECs), among which we distinguish large-cell NECs (LC-NECs) and small-cell NECs (SL-NECs), usually with Ki67 > 55%. These classifications are not valid for extra GEP-NENs [25,26,27,28].

### 1.2. Types of Hormonally Active Neuroendocrine Neoplasms (NENs)

The clinical symptoms of neuroendocrine neoplasms of the digestive tract depend on the location of the tumor’s primary site, and the amount of secreted peptide hormones and biogenic amines [8]. The most frequent initial symptom in patients with small intestinal NENs (siNENs) is abdominal pain resembling irritable bowel syndrome, but the great majority of GEP-NEN patients present symptoms characteristic of advanced cancer, such as anorexia, weight loss and fatigue; less than 5% of NETs are connected with a hormonal syndrome [24,25,26,27]. In hormonally active tumors, symptoms include hypoglycemic syndrome, carcinoid syndrome, Zollinger-Ellison syndrome, watery diarrhea-hypokalemia-achlorhydria syndrome (WDHA), and glucagonoma [28].

Presented below are classifications of NENs made on the basis of secreted substances; this is valid only for functioning forms that represent a minority of NENs.

A carcinoid is a hormonally active tumor originating from the central section of the digestive tract, characterized by the possible release of serotonin and other biologically active substances (kinin, tachykinin, dopamine, histamine, and prostaglandin) into the system’s circulation, causing symptoms characteristic of carcinoid syndrome with accompanying liver metastases [29,30]. Carcinoid syndrome affects 4–10% of patients with small intestine NET location. The symptoms are present when liver metastasis occurs, and the produced serotonin, un-metabolized by hepatocytes, permeates directly into circulation. According to ENETS Consensus Guidelines Update 2016, carcinoid syndrome could be present in 20–30% of patients with metastases [26,31]. The slow growth of the tumor contributes to delayed diagnosis due to an asymptomatic course for many years. Carcinoid syndrome may manifest through explosive and watery diarrhea, present up to 30 times a day, which occurs for about 80% of carcinoid patients. The second most common symptom is paroxysmal skin flushes, from salmon-colored up to dark red, which affects about 85% of patients and usually occurs in the upper parts of the body (face, neck or chest). Common triggers include tyramine-containing foods (bananas, chocolate, blue cheese, red wine), alcohol, and stress. Carcinoid syndrome can be related to other symptoms: stomach aches, dizziness, telangiectasia, pellagra, tiredness, and sometimes the impairment of cognitive functions [32]. Hedinger syndrome occurs in over half of patients with carcinoid syndrome and can be the main cause of death due to right-side heart failure because of morphological changes and mechanical damage to the right heart valve apparatus due to un-metabolized serotonin [33,34]. For this reason, every patient with carcinoid syndrome requires cardiology consultation with echocardiography [26].

Insulinoma, originating from β cells, is the most frequent hormonally active pancreas NET, overproducing insulin and leading to hypoglycemia and hypoglycemic syndrome. The symptoms of hypoglycemia occur suddenly and paroxysmally in the morning due to significant fasting and after intense physical activity; they are accompanied by sweating, paleness, restlessness, shivering, palpitations, and hypersalivation [35]. The next stage of hypoglycemia includes psychomotor and concentration disorders, resulting in the loss of consciousness. Typical for insulinoma is a significant gain of body mass, mainly due to strong hunger caused by hypoglycemia, resulting in excessive caloric intake [34,35].

Gastrinoma is a gastrin-producing NET usually located in the pancreas or the front wall of the duodenum. In about 60% of patients, the tumor is malignant, resulting in metastasis, most often to the nearby lymph nodes and the liver [36,37]. Gastrin overproduction leads to overgrowth of parietal cells and increased secretion of stomach acid, causing stomach and duodenum ulceration. Furthermore, it deactivates pancreas enzymes, resulting in incorrect fat absorption and causing diarrhea. Severe ulcer disease combined with diarrhea in gastrinoma patients is known as Zollinger-Ellison syndrome (ZES). ZES patients typically suffer from stomach pain, vitamin B_12_ absorption disorders, a loss of body mass, colic, and kidney stones [36,37].

VIPoma is a NET producing vasoactive intestinal peptide (VIP). VIP is a neurohormone released by the central nervous system, intestines, pancreas, the respiratory tract, and the urogenital tract. VIP regulates the activity of smooth muscles, dilates blood vessels, and is responsible for water and electrolyte secretion by the digestive tract and inhibition of stomach acid secretion. Typically, the symptoms of excessive VIP secretion include watery diarrhea, hypoglycemia, achlorhydria, sometimes hypercalcemia or hypophosphatemia, and metabolic acidosis. The diarrhea volume usually exceeds 700 mL/24 h, and in 70% of cases can reach 3000 mL a day. Patients describe it as odorless, with a tea-like color. VIPoma can be connected with symptoms like lethargy, nausea, vomiting, and the weakening of muscles, as well as contractions that occur as a result of dehydration and hypoglycemia [34,38].

Glucagonoma originates from the α cells of pancreatic islets, which produce glucagon [39]. Its clinical image includes symptoms such as necrolytic migratory erythema (NME) (82%), usually located in the area of the lips and sexual organs, diabetes (80%), body mass loss (90%), a low level of zinc, niacin deficiency, abdominal pain, diarrhea, normochromic normocytic anemia (61%), and episodes of glossitis. Patients have thinning hair and dystrophic nails. Glucose intolerance in glucagonoma syndrome usually occurs proportionally to the size of the tumor. The concentration of glucagon in the plasma when fasting is higher in the group of patients with liver metastasis than in patients without accompanying metastasis. Liver metastasis reduces the ability of the liver to metabolize glucagon, increasing its concentration in peripheral blood [40].

Somatostatinoma originates from the cells of pancreatic islets which produce somatostatin. Somatostatin is an inhibitor of numerous secretory hormones, such as insulin, glucagon, gastrin, secretin, and motilin. Apart from strong inhibition, it has a direct influence on many target organs. It influences the activity of the intestines in terms of the absorption of nutrients, mainly fats and calcium. By stimulating prostaglandins, it slows down the secretion of stomach acid. The dominating illnesses in the clinical image of somatostatinoma are gallbladder stones, fat stools, body mass loss, and mild diabetes, which form somatostatinoma syndrome [34]. The most common location for the metastasis of this tumor is the liver, then lymph nodes and, lastly, the bones. The total removal of the tumor is usually very effective in the therapy of this illness [41,42]. It is worth mentioning that the presence of pancreatic neuroendocrine tumors (pNET) can occur as a part of inherited syndromes like multiple endocrine neoplasia type I (MEN 1), which could be responsible for 20–30% of gastrinomas, <5% of insulinomas, and rarely functional pNETs. Uncommon causes of pNETs include other inherited syndromes like von Hippel Lindau disease (VHL), neurofibromatosis type 1 and tuberous sclerosis [43].

## 2. The Treatment of Neuroendocrine Neoplasms

The treatment of choice is a surgical intervention with the curative or palliative aim, depending on the location and histopathology of the tumor. To qualify for surgery, patients have to be in generally good condition, with a tumor limited only to the primary site and the nearby lymph nodes. Patients with potentially resectable liver metastasis also qualify for the intervention [44,45]. Unfortunately, due to the presence of late clinical symptoms, NENs are usually diagnosed at advanced stages of the illness. This is why, in most cases, it is impossible to fully eliminate the changes [22].

Somatostatin analogues (SSAs) are an important part of NEN therapy and can be administered in neuroendocrine neoplasms (long-acting SSAs) as well as in a carcinoid crisis (short-acting SSAs). In treatment, we can use two types of SSA: octreotide and lanreotide. SSAs inhibit the secretion of many hormones, fulfill immunological, cytotoxic and cytostatic functions, and in specific conditions they can also be apoptotic through their direct influence on the somatostatin of tumor cells receptors (SSTR). In an indirect way, they lead to the inhibition of tumor mass factors, the proliferation of lymphocytes, and immunoglobulin synthesis. From an oncological point of view, the anti-proliferating effect of somatostatin analogues is the most important aspect, as it slows down the development of the illness and reduces the size of the tumor. It influences the digestive system in a multi-directional manner, slowing down the blood flow of visceral vessels as well as intestinal motility and transport. Most importantly, it inhibits the secretion of pancreatic and intestinal hormones. SSAs play a particularly important function in patients with hormonally active GEP-NENs. After they are administered, the symptoms associated with excessive secretion of biogenic substances are alleviated, improving the quality of life [44,45,46,47]. In the PROMID study, it was proven that long-acting repeatable octreotide acetate (octrotide LAR) significantly lengthens the time of tumor progression in patients with functionally active and inactive metastatic midgut NETs, and, a few years later, in the CLARINET study, it was shown that lanreotide therapy was associated with significantly prolonged progression-free survival (PFS) among patients with metastatic NETs of grade 1 or 2, with Ki-67 < 10% [48,49]. In the TELECAST study, it was proven that in patients with carcinoid syndrome not adequately controlled by SSA therapy, telotristat etiprate, an inhibitor of tryptophan hydroxylase, can be used to limit the synthesis of serotonin [50].

In patients in an advanced stage of the disease, as well as in those with relapses after primary therapy and who did not undergo full surgical treatment, other types of therapy, like peptide receptor radionuclide therapy (PRRT), Tyrosine Kinase Inhibitor (TKI), mTOR inhibitors, or chemotherapy, can still be used [24].

PRRT is another type of GEP-NEN treatment based on “a combination of somatostatin analogues with yttrium or lutetium isotopes, and the cytotoxic factor is the ionizing radiation of the isotope” [51]. Radionuclide therapy seems to be a good method when patients intensely accumulate the marker at each neoplasm site of a small size, which can be used to achieve total remission, or at least a reduction in the neoplasm’s mass [52]. In the presence of liver metastases by GEP-NEN, as a form of palliative treatment, radiofrequency ablation (RFA), trans-arterial embolization (TAE), and trans-arterial chemoembolization (TACE) can be offered [53].

Multi-target tyrosine kinase inhibitors (MTKIs), such as axitinib, cabozantinib, famitinib, lenvatinib, nintedanib, pazopanib, sorafenib and sulfatinib, represent a new approach to NEN treatment [54]. Sunitinib malate has been approved by regulatory agencies for pancreatic NENs [55]. Sunitynib is an oral multi-targeted inhibitor of various receptor tyrosine kinases that leads to a decrease in angiogenesis, growth, proliferation, and metastatic spread [56].

According to the RADIANT-3 and RADIANT-4 studies, the mTOR inhibitor ewerolimus has an established place in the therapy of advanced and progressive pancreatic NETs and non-functional lung and gastrointestinal NETs [57,58].

When metastasis is present or in case of disease progression in patients with NETs chemotherapy can be used. Capacitabine and Temozolemide (CAPTEM) shows significant activity in patients with metastatic well-differentiated pancreatic NETs [59]. At the same time, CAPTEM presents significant activity in patients with metastatic grades 2 and 3 pancreatic and non-pancreatic NETs with manageable toxicity. Systemic combined chemotherapy like cisplatin + etoposide, streptozocine + 5-fluorouracil, streptozocin + doxorubicin, leucovorin + 5-fluorouracil + oxaliplatin (FOLFOX) or leucovorin + 5-flurouracil + irinotecan (FOLFIRI) has been designed to treat NEC patients according to primary tumor localization [60,61].

In the randomized clinical trials (RCT) of neuroendocrine tumors, 22 different therapy strategies were compared, stating that there are a number of effective therapies with different safety profiles available to patients, suggesting an overall superiority of combination therapies [62]. For patients with advanced NETs as a best second- and third-line treatment, respectively, to progression-free survival (PFS), PRRT, SSA + bevacizumab, and SSA + interferonalfa should be considered [63].

In the absence of an optimal treatment strategy, other methods should be found. There are several future potential NEN therapies, such as immunotherapy (programmed death ligand 1, cytotoxic T-lymphocyte antigen-4 blockers) and somatostatin-dopamine multi-receptor chimeras [64].

## 3. The State and Method of Nutrition with Reference to the Risk of Cancer

Epidemiological studies strongly suggest that BMI and especially visceral fat accumulation, decreased physical activity, and unhealthy diets are key elements in the pathogenesis and prognosis of many common cancers. The phenomenon known as “the obesity paradox” suggests a potentially protective effect in patients with overweight or slight obesity, increasing their survivability after diagnosing a neoplastic disease. [65].

The accumulation of multiple DNA mutations in critical genes (oncogenes or tumor suppressor genes) of particular cells, if not properly controlled through the induction of senescence or apoptosis, can lead to uncontrolled cell proliferation and the progressive transformation of cells into highly malignant tumor cells [66]. Calorie restrictions without malnutrition are the most potent and reproducible physiological intervention for increasing lifespan and protecting against cancer [67].

Not many nutrients have a cause and effect relation with cancer, some of them include: fried, smoked or roasted red meat, food contaminated with aflatoxin, preserved salty meals, excessive alcohol consumption [67,68]. The risk of cancer can also be reduced by introducing a diet rich in plant food (e.g., vegetables, beans, fruit and wholegrain products) and by limiting the consumption of animal fat, meat and fatty dairy products [68].

One such diet is the Mediterranean diet (MD), which could influence the reduction of the aggressiveness of different tumor types and tumor size [16,69]. The diet plays an important role in the initiation, promotion, and progression of cancers [70]. Vegetables and fruits are important sources of a wide variety of micronutrients and other bioactive compounds, including antioxidants, vitamins, folates, carotenoids, glucosinolates, indoles, isothiocyanates, protease inhibitors, and phytochemicals, such as lycopene, phenolic compounds, and flavonoids, which have been demonstrated to exhibit anticancer properties [70,71]. All these compounds may act against cancer through different mechanisms, including their antioxidant, anti-mutagenic, and anti-proliferative properties. Furthermore, there is a connection between obesity and the increased risk of endometrium, breast, colon, esophagus, kidney, pancreas, gallbladder or liver cancers [72]. Obesity and an excess of fat tissue resulting from a chronic energy imbalance are associated with the increased risk of oxidative stress. Consequently, there are disorders of lipid and carbohydrate metabolism, insulin resistance, systemic inflammation, changes in hormone levels and growth factor concentrations which have a key role in the pathogenesis of many neoplasms.

### 3.1. Dietary Recommendations for NEN Patients

Nutrition care plans are an integral part of the multidisciplinary management of patients with NETs. Nutritionists with expertise in NETs can provide dietary approaches to improve the quality of life and nutritional status during therapeutic modalities used for patients with NETs and particular in palliative care [73]. Unfortunately, there are not enough registered physicians and dieticians who have expertise in the nutritional management of NETs [74]. Factors such as unhealthy diets, tobacco, alcoholism, infections and occupational exposures contribute to the formation of neoplasms [75]. The need for consistent dietary guidelines for NEN patients and collaboration with nutritionists in multidisciplinary healthcare teams in NET management have been emphasized by other authors [69,70,71,72,73,74,75,76]. Recommendations for a healthy diet are based on the 2015–2020 Dietary Guidelines Advisory Committee for patients with newly diagnosed asymptomatic NETs [77]. Therefore, a diet with five servings of vegetables and fruits, including legumes and with meat restrictions, will be the best solution for newly diagnosed and asymptomatic patients [78,79]. However, the diet will depend on the symptoms of each patient, the stage of the disease, the type of therapeutic management, and the individual’s nutritional status. The best diet for a NEN patient is, therefore, an individualized diet. Moroever, dietary recommendations in neuroendocrine neoplasms should take into account the excessive production of hormones, the source of which could be endogenous or exogenous. Furthermore, an excess of biogenic amines in the body often leads to the occurrence of specific symptoms, such as diarrhea, nausea, vomiting, and metabolic disorders in the form of hypoglycemia and hyperglycemia, contributing to malnutrition and the general weakening of the patient [80,81,82].

### 3.2. The Causes of Diarrhea in NEN Patients and Dietetic Modifications

The presence of diarrhea in NEN patients may result from various factors, including the metabolism of hormonally active NEN, biogenic amines, drugs and niacin deficiency.

Diarrhea occurs independently of the consumed meal. It can occur after the meal, especially after the consumption of large amounts and fatty products, but it can also occur after fasting and at night. In the case of some neoplasms (somatostatinoma and, less often, gastrinoma), diarrhea is associated with impaired digestion or the absorption of fatty acids in the digestive tract. Lastly, diarrhea can be caused by surgical intervention in the area of the intestine, which leads to a direct loss of absorbency [41,42,83].

**Figure 1:**
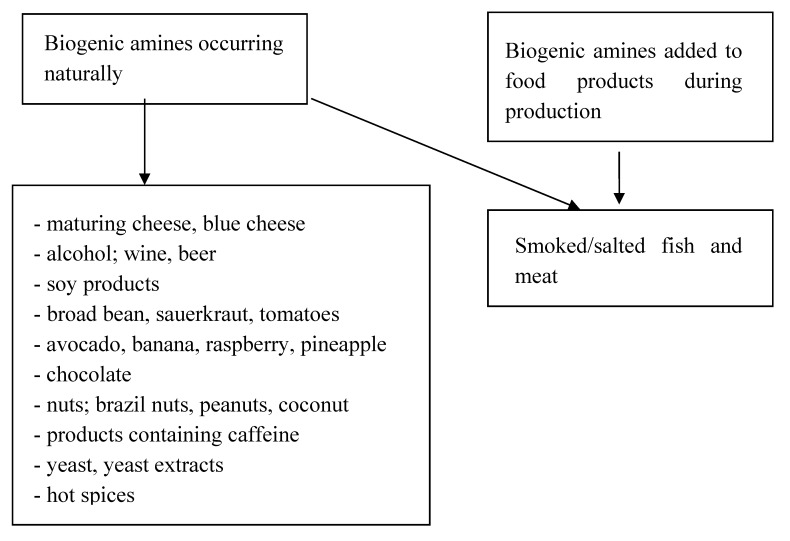
Presence of amines in food products.

Biogenic amines are nitrogen compounds that are present in products in two forms: naturally, due to synthesis by plants, animals and microorganisms, or as an additive during production, in the form of preservatives [84]. Their synthesis occurs as a result of the breakdown of peptides and proteins. They then undergo further transformations, creating new, different amines. Physiologically, they participate in numerous processes; they are a source of nitrogen and they are precursors of the synthesis of hormones, nucleic acids, and proteins [85]. The synthesis of amines in food depends on the availability of appropriate amino acids and bacteria, as well as on the environmental conditions, which determine the correct activity of enzymes and bacterial growth. Their activity is observed in numerous food products, including drinks. Figure 1 shows products that include significant amounts of amines, as well as products that should be eliminated from the diet with some GEP-NENs [86,87].

The most frequent causes of symptoms are histamine, tyramine and putrescine. Patients with a genetic deficiency in diamine oxidase (DAO), as well as those that take medicine belonging to the group of monoamine oxidases, are particularly susceptible to the negative effects of amines [85,86]. Histamine is formed from histidine with the co-participation of histidine decarboxylase enzyme [87]. Mastocytes, basophils, and enterochromatophilic cells of the digestive tract are the most significant cells that produce endogenic histamine. They have the ability to store this amine in cytoplasmic granulation and, in the subsequent stage, to release the amine into blood circulation through immunological and non-immunological stimuli. Histamine metabolism proceeds in two ways [88]. The first features methylation into N-methylhistamine via N-methyltransferase (HNMT). Thanks to this enzyme, histamine can be metabolized only intracellularly. The second method is oxidation into imidazoleacetic acid thanks to the activity of diamine oxidase (DAO). Disease of the digestive tract, as well as the activity of alcohol and some medicines, can be the cause of a secondary shortage of DAO. The role of histamine is mainly based on the extracellular metabolism of the described biogenic amine, so it is reduced after the consumption of products that are a rich source of histamine. Exogenic sources of histamine are products that take a long time to mature and ferment, such as baker’s yeast, red and white wine, beer, champagne, kefir, blue cheese, cheese spread, yellow cheese, prosciutto, salami, highly processed cold meat, smoked fish, avocado, spinach, eggplant, sauerkraut, ketchup, and various spices and herbs [83,84,89,90]. Tyramine can be the cause of reddening, which is one of the most common clinical carcinoid symptoms. It is an aromatic monoamine whose systematic name is 4-hydroxy-phenethylamine. Numerous studies have demonstrated that tyramine is the most common biogenic amine in cheese. It is found in the highest content in veined blue cheese, such as gorgonzola or roquefort. The amount varies in specific parts of the cheese, with the highest content in the external part. The primary producers of tyramine in cheeses are Gram-positive bacteria, such as Lactobacillus, Enterococcus, Leuconostoc, Lactococcus and Carnobacterium [88,89]. Moreover, it is also common in fermented foods, such as soy sauce, shrimp spread, marmite, eggplant, spinach, sauerkraut, sausages, ham, smoked fish, anchovies, sardines, beer, wine, and chocolate. The characteristic term associated with tyramine poisoning is the “cheese effect”, which is highlighted by a hypertensive crisis in blood pressure. There have been numerous cases of death resulting from myocardial infarction or stroke as a consequence of consuming food products with significant amounts of tyramine [89].

Out of all of the symptoms of GEP-NENs, the most common are skin reddening and diarrhea, the latter of which leads to dehydration and electrolyte disturbances [2,5,90,91]. They appear as a result of the promotion via the secretion of hormones, peptides, and amines, and the excessive secretion of liquids to the intestinal mucosa [91,92]. Dietary treatment recommended avoiding hot, seasoned, fatty and overly large meals when suffering with diarrhea. One should consume meals that include proteins, mainly lean products of meat, poultry, curd cheese, eggs, and yoghurt. As a source of carbohydrates, starch products such as rice and finely ground oats are recommended. Simple sugars (glucose and fructose) should be eliminated from the diet because they strengthen fermentation processes. It is recommended to exclude lactose and saccharose for a few days. The following products are rich in the aforementioned sugars: jams, honey, candy, and apple and grape juice (which should be particularly avoided). Vegetables should be mild. They should not cause excessive production of flatulence and are best served shredded and boiled. The best are those with high amounts of pectin, such as pumpkin and carrots, and when it comes to fruit, the best ones are apples and bananas. Meals should be served with plant fats to strengthen the energy properties of the diet and reduce the glycemic index. It is also crucial to supply liquids (potassium water in particular) to avoid dehydration. It is recommended to consume two liters per day [93,94]. One should eliminate liquids that include caffeine and strong tea because they do not hydrate well enough and they are rich in biogenic amines [95,96]. There are only a few references to the use of enteral nutrition in the clinical guidelines of patient management with oncology treatment-related diarrhea. Although no data is available for patients with NENs, it appears that the inclusion of oligomeric enteral nutrition formula in patients with diarrhea and malnutrition may be justified [97]. The essential element will be to determine the functional capacity of the patient’s intestine and nutritional status. Therefore, nutrition in this case may be based on typical dietary recommendations, through supplementation with oral oligomeric enteral nutrition [98] along with full enteral nutrition with oligomeric formula, up to potentially complete parenteral nutrition [99]. When it comes to the share of dietary supplements, products with high osmolality should be avoided [100], and supplements could even be included in cases of severe diarrhea or severe malnutrition [101]. It is important to be cautious when using nutraceuticals or other dietary supplements because of the possibility of disrupting chemotherapy. However, due to the deficits observed in these patients, it is important to conduct chemotherapy [66,102,103].

### 3.3. Procedures to Follow in the Case of Constipation in NEN Patients

In some cases, constipation can appear in NEN patients. It is usually accompanied by abdominal distension and flatulence [104]. The main cause is the ileus, or a side effect of the applied pharmacological therapy. The excessive secretion of catecholamines by the tumor can reduce the peristaltic activity of the digestive tract, leading to chronic constipation [34]. The undertaken nutritional intervention should cover the increased supply of liquids in the form of mineral water, juice, and chamomile or fennel tea. It is recommended to consume products with high amounts of insoluble dietary fiber such as Graham bread, bran bread, thick groats, or oatmeal. Inulin in particular has properties that support the struggle with constipation [105]. It has also been found that the low fermentable oligosaccharides, disaccharides, monosaccharides, and polyols (FODMAP) formula improves diarrhea and nutritional status in hospitalized patients [106]. Cruciferous and bulbous vegetables are not recommended due to causing excessive flatulence [85]. Moreover, in patients with chronic constipation, dysbiosis is observed in the area of the small intestine, with Clostridium and Enterobacteriaceae as the dominating bacteria. This is why it is so important to include probiotic bacteria that produce short-chain fatty acids (*Lactobacillus* and *Bifidobacterium*) in order to reduce the pH of the intestine, to stimulate intestinal motility and to accelerate the transit of stool [106,107]. Regular physical activity is also beneficial [65,66]. For a better understanding of the individual nutritional approach to the patient with NEN, considerations are presented in Table 1A.

### 3.4. Nutrition that Takes into Account the Hormone Activity of NEN

When analyzing all nutritional aspects, it is also important to consider the hormone activity of NEN, which has an influence on metabolic changes in patients.

As a result of the excessive production of insulin, there is a reduction in the level of glucose, which can, in serious cases, be the cause of death. The aim of dietetic therapy is to prevent long fasting between meals via the frequent consumption of small portions of food during the day and at night. A high-protein diet is also important as glucose can be metabolized by the organism for a longer period of time, and its secretion to the circulation is slower. Thanks to that, the risk of secondary hypoglycemia decreases, and the increase in body mass, which is characteristic of insulinoma, is unnoticeable. Products with a low glycemic index and complex carbohydrates maintain the level of glucose at a stable level, preventing post-meal hypoglycemia. When the level of glucose in the blood decreases, it is important to immediately supply carbohydrates with a high glycemic index like fruit juice, because they are quickly absorbed [39,40,41].

The reverse effect, hyperglycemia, usually develops secondarily, most often in patients predisposed to diabetes. In the case of NEN, hyperglycemia develops due to the direct influence of the tumor mass on the pancreas, reducing the amount of insulin, as a result of surgical treatment, and also due to pharmacological treatment with a somatostatin analogue (SSA). This effect of treatment with a somatostatin analogue may be caused by impaired fasting glucose (IFG), impaired glucose tolerance (IGT), and diabetes mellitus (DM). These patients should have a healthy, balanced diet, based on products with a low glycemic index. Food products should be combined with fats, which will help keep glucose levels constant. High consumption of fiber should also be considered [15,96].

**Figure 2:**
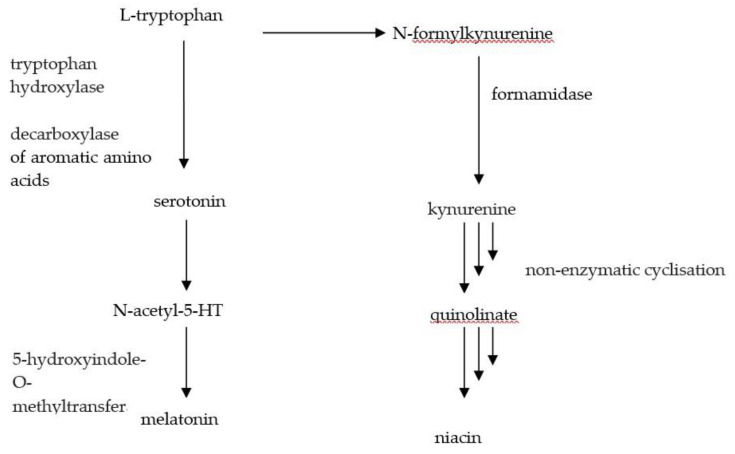
Transformation of tryptophan.

Another issue that has to be taken into account is the common niacin deficiency and risk of pellagra, which occurs in patients with carcinoid syndrome [108]. Vitamin B_3_ deficiency results from the increase of the metabolism of tryptophan into serotonin (Figure 2).

The deficiency leads to skin inflammation, diarrhea and mental disturbance, which can lead to death in severe cases if left untreated [109]. The skin becomes rough, fractures easily, and discoloring occurs. The most noticeable changes are to the face, neck and hands. Patients should be supplied with food rich in this vitamin, such as liver, fish, meat, yeast, wheat bran and the seeds of leguminous plants. In smaller amounts, niacin is also found in fruit and vegetables, bakery products, and milk [57]. When the patient is unable to cover their needs through consumed food, supplementation of this vitamin should be taken into consideration, supplying from 25 to 50 mg/day [16].

The disorders of digestion and/or the absorption of fatty acids that cause fatty diarrhea may be the result of gastrinoma or—less frequently—somatostatinoma. This is why it is important to reduce the supply of fats in the diets or to propose a substitution of pancreatic enzymes [110]. For a better understanding of the individual nutritional approach to the patient with NEN, considerations are presented in Table 1B.

### 3.5. A Diet for NEN Patients under the Risk of Malnutrition and/or Cachexia

Two studies have indicated that as many as one in four NET patients are malnourished, as assessed using the Malnutrition Universal Screening Tool and Subjective Global Assessment (SGA) tool [111]. Screening for malnutrition should be a part of routine care in every GEP-NEN patient [98]. Malnutrition has substantial negative consequences for cancer patients including increased mortality, poorer quality of life, and increased health care costs [112]. Malnutrition, which leads to the devastation of the body, is often associated with cancer cachexia, which is characterized by a loss of fat and muscle mass. The main cause of malnutrition is limited food consumption by the patient, which might amplify the symptoms associated with the treatment, such as nausea, vomiting, inflammation of the mucous membrane, abnormal absorption, anorexia, tiredness, and pain [113]. The reasons for the development of cachexia are (among other) metabolites produced by the tumor, which can cause anorectic effects in the center of hunger and satiety located in the brain. Another factor is systemic inflammation, which amplifies hypermetabolism, body mass loss, and tiredness. There are several studies reporting malnutrition in NENs. The range of reported malnutrition is 4.9%–38% in the course of progressive disease [114,115]. Omega-3 fatty acids have a positive effect on the treatment of neoplastic cachexia [116]. To cover the increased caloric needs, the patient’s diet should include an appropriate amount of all nutrients: proteins, fat, carbohydrates, vitamins and minerals. Sometimes, apart from meals, it will also be important to supply patients with dietary supplements that include necessary nutrients [95]. Arginine regulates the production of NO in cancer and thus in might support the development of anti-cancer drugs that target this key metabolic pathway [117]. The diet should prevent body mass loss, lead to the reconstruction of tissues, and improve the way the patient feels. It is recommended they consume meals more frequently, but in smaller amounts. It is also recommended patients eat snacks between meals. It is not recommended to drink between meals due to the excessive dilution of gastric juice, which disturbs digestive processes and increases food volume. In patients that have problems with the consumption of meals or in severely malnourished patients, it is recommended to introduce enteral nutrition or parenteral feeding [7]. The content of the applied mixture should be individually adjusted to the needs of the patient, taking into consideration his illnesses and providing all the necessary nutrients [118].

## 4. Dietary Care Taking into Account Pharmacotherapy

It is important to consider the interaction of some medicines with food and the changes in the secretion by some organs, such as the pancreas. During the investigation it was determined that the treatment with everolimus or sunitinib may pose a risk for patients because there is an interaction with food (grapefruit, camomile, cranberry, garlic, ginseng, green tea extract, pepper, resveratrol and soy) associated with the inhibition of P450 (CYP) 3A pathway, which may lead to the toxicity of the medicine. Moreover, high-fat meals are inhibitors of tyrosine kinase [66]. Furthermore, temozolomide should not be supplied with food because fat—by changing the pH of the stomach—inhibits CYP P450. It has also been demonstrated that somatostatin analogues that are common in the treatment of advanced well-differentiated NEN may lead to exocrine pancreatic insufficiency in some patients [119]

For a better understanding of the individual nutritional approach to the patient with NEN, considerations are presented in Table 1C.

## 5. Summary

Neuroendocrine neoplasms of the digestive tract, through the secretion of hormones, peptides and biogenic amines, cause various symptoms that can be reduced to improve the quality of life through a balanced diet and physical activity. The inclusion of low glycemic index products in the diet prevents the abrupt decrease and fluctuations in the level of glucose in the blood. This illness may result in the limitation of the consumption of some nutrients, leading to the development of nutritional deficiencies, excessive body mass loss and, eventually, malnutrition. Cachexia can be observed in some NEN patients. It can occur as the result of the tumor’s production of metabolites that have an anorectic effect on the center of hunger and satiety in the brain. This is why enteral nutrition support and parenteral feeding should be considered in these patients. The frequent presence of diarrhea can be amplified by the consumption of biogenic amines. Therefore, patients that suffer from this problem should eliminate the following products from their diet: smoked fish and meat, soy products, avocado, raspberries, pineapples, chocolate and nuts. The carcinoid syndrome is characterized by the deficiency of niacin that results from the increased metabolism of tryptophan into serotonin, which can explain frequent changes in moods. A well-balanced nutritional plan not only supports the struggle against the illness, but it also eliminates the side effects of therapies, improving the quality of life.

### 5.1. Structure of the Underlying Research

The present narrative review evaluates the above-mentioned topics by considering the literature published up to 31 December 2019. A literature search was conducted utilizing the PubMed and Scopus databases. The terms used were: neuroendocrine tumors, neuroendocrine neoplasms, gastroenteropancreatic neuroendocrine neoplasias, and biogenic amines. The passwords were checked on terms: neuroendocrine tumors, neuroendocrine neoplasms, gastroenteropancreatic neuroendocrine neoplasias, and biogenic amines. These terms were combined with diagnosis, treatment, nutrition, diarrhea, constipation, and nutrition assessment. Studies that were not in English, letters to editor, and abstracts to conferences were excluded. All included studies were screened and discussed by the authors until a general consensus was reached.

#### Ethical Approval

This article does not contain any studies with human participants or animals performed by any of the authors.

## Figures and Tables

**Table 1:** (**A**) Proposed dietary care solutions for patients with Nuroendocrine neoplasm (NEN) according to the patient’s nutritional status (BMI). (**B**) Proposed solutions for the dietary care of patients with NEN, taking into account NEN hormone activity. (**C**) Sample pharmacotherapy of NEN patients taking into account interactions with food.

(**A**) Proposed dietary care solutions for patients with NEN according to the patient’s nutritional status (BMI).		
Nutritional status (BMI)	Symptoms	Dietary care solutions
>30 Obesity, visceral fat accumulation	No persistent bothersome symptoms	Anti-neoplastic diet (based on the high quantity and diversity of plant products) or Mediterranean and additionally reduction diet [69,70,71,74,75,76,77,78,79] Regular physical activity adjusted to the patient’s capabilities [77,78,79].
	Severe diarrhea with progressing reduction of body mass	Consider supplementation, especially with omega 3 [94,116]; diet that includes a reduced amount of fiber, legumes and brassicas until symptoms decline [15,80,94]; Elimination of biogenic amines [87,88,92]; Electrolyte supplementation [91]; Potassium supplementation (hypokalemia) [15]; Avoid hot, very spicy, fatty and large meal portions [66]; Consume meals that include proteins, mainly lean poultry, cottage cheese, eggs and yoghurt; Carbohydrates: rice and finely ground oats, pumpkin, carrot, bananas, apples [66]; Exclude lactose, saccharose, fructose and glucose [15,66,93].
	Constipation	Anti-neoplastic, Mediterranean diet [69,70,71,74,75,76,77,78,79] high in fiber with inulin [15,102], consider probiotics therapy: *Lactobacillus acidophilus* and *Bifidobacterium lactis* [106]; Increased supply of liquids (mineral water with lemon, aloe, additionally drank in the morning) [34]; Regular physical activity, physiotherapeutic massage [74,75,76].
	Disturbed carbohydrate metabolism	Low glycemic index diet with limited amounts of fruit (glucose, fructose, saccharose), supplemented with MUFA and PUFA [66,67,68,69,70,71,72,73,117]; Regular physical activity [77,78,79].
* 26–29.9 overweight Visceral Fat accumulation <26	No chronic, irritating symptoms	Anti-neoplastic, Mediterranean diet. Perhaps consider a reduction diet if the patient’s diet did not decrease recently due to the intense course of the disease [69,70,71,74,75,76,77,78,79]; Regular physical activity [77,78,79].
	Irritating diarrhea with progressing reduction of body mass	Procedures the same as in the case of diarrhea >30 BMI.
	Constipation	Procedures the same as in the case of constipation >30 BMI.
	Disturbed carbohydrate metabolism	Procedures the same as in the case of disturbed carbohydrate metabolism >30 BMI.
26–22/23 **	No chronic, irritating symptoms	Anti-neoplastic, Mediterranean diet according to needs of the body [69,70,71,74,75,76,77,78,79] Regular physical activity [77,78,79].
	Irritating diarrhea with progressing reduction of body mass	Procedures the same as in the case of diarrhea >30 BMI.
	Constipation	Procedures the same as in the case of constipation >30 BMI.
	Disturbed carbohydrate metabolism	Procedures the same as in the case of disturbed carbohydrate metabolism >30 BMI.
<22/23 ** At the risk of malnutrition	No chronic, irritating symptoms	Anti-neoplastic, Mediterranean diet [69,70,71,74,75,76,77,78,79] Stimulation of tissue reconstruction, e.g., through the incorporation of industrial diet preparations that additionally feature arginine [117].
	Irritating diarrhea with progressing reduction of body mass	Incorporation of oligomeric formula of enteral nutrition in patients with diarrhea and progressing malnutrition [97,98]; Potentially–full parenteral nutrition [101]; Diets with reduced osmolarity [100]; Electrolyte supplementation [91]; Incorporate multi-element supplementation that includes omega-3 [95].
	Cachexia	Enteral nutrition and parenteral nutrition, omega-3 supplementation [102], multi-element supplementation [89,95].
(**B**) Proposed solutions for the dietary care of patients with NEN taking into account NEN hormone activity		
NEN	Symptoms	Nutrition
Carcinoid	Increased metabolism of tryptophan into serotonin/spastic diarrhea	Supplementation of niacin deficiency (vitamin PP), supplementation 25–50 mg/day [16]; Include the consumption of fish, meat, bran and the seeds of legumes [106,107]; Regular physical activity after the earlier analysis of the heart using echocardiography [72].
Gastrinoma	Increased gastric acid synthesis and inactivation of pancreatic enzymes. Disorders of digestion and/or absorption of fatty acids = fatty diarrhea	Consume meals that include fats, mainly lean poultry, cottage cheese, eggs and yoghurt; Carbohydrates: rice and finely ground oats, pumpkin, carrot, bananas, apples; Limit fats or include pancreas enzymes’ substitution [88,89]; Regular physical activity [72].
Somatostatinoma	Inhibition of the exocrine pancreatic function/steatorrhea	Procedures the same as in the case of gastrinoma.
Vipoma	Water and electrolyte secretion by the digestive tract and inhibition of stomach acid secretion/secretory diarrhea	Special care for hydration and electrolyte management [91].
Glucagonoma	Disturbed carbohydrate metabolism; glucagon overproduction; impaired fasting glucose/impaired glucose tolerance/diabetes	Low glycemic index diet with the limitation of fruit; Prevention of long fasts between meals during the night break; Regular physical activity [72].
Insulinoma	Disturbed carbohydrate metabolism insulin overproduction/hypoglicemia	In the case of frequent hypoglycemia in insulinoma, the supply of carbohydrates with a high glycemic index, e.g., fruit juice [34,35,36,37,38,39,40,41]; Prevention of long fasts between meals during the night break.
(**C**) Sample pharmacotherapy of NEN patients taking into account interactions with food		
Medicine	Influence	Food
Everolimus, sunitinib [66]	P450 (CYP) 3A4 inhibition	Exclude for the diet: grapefruit, camomile, cranberry, garlic, ginseng, green tea extract, pepper, resveratrol and soy
Sorafenib	Inhibitors of tyrosine kinase	High fat meals
Capecytabine	is unstable under strongly acidic conditions	should be administered with a meal (up to 30 min after a meal)
Temozolomide [66]	CYP P450 inhibition through stomach pH	Not to be supplied together with food (on empty stomach)
Long-acting somatostatin analogues [110,119]	Exocrine pancreatic insufficiency	Include the substitution of pancreatic enzymes

* higher survival rate [65]; ** women/men.
